# Larval food quantity affects the capacity of adult mosquitoes to transmit human malaria

**DOI:** 10.1098/rspb.2016.0298

**Published:** 2016-07-13

**Authors:** Lillian L. M. Shapiro, Courtney C. Murdock, Gregory R. Jacobs, Rachel J. Thomas, Matthew B. Thomas

**Affiliations:** 1 Department of Entomology, The Pennsylvania State University, University Park, PA 16802, USA; 2 Center for Infectious Disease Dynamics, The Pennsylvania State University, University Park, PA 16802, USA; 3 College of Veterinary Medicine, University of Georgia, Athens, GA 30602, USA; 4 Odum School of Ecology, University of Georgia, Athens, GA 30602, USA

**Keywords:** extrinsic incubation period, vector competence, vectorial capacity, *Plasmodium falciparum*, vector borne disease, *Anopheles*

## Abstract

Adult traits of holometabolous insects are shaped by conditions experienced during larval development, which might impact interactions between adult insect hosts and parasites. However, the ecology of larval insects that vector disease remains poorly understood. Here, we used *Anopheles stephensi* mosquitoes and the human malaria parasite *Plasmodium falciparum,* to investigate whether larval conditions affect the capacity of adult mosquitoes to transmit malaria. We reared larvae in two groups; one group received a standard laboratory rearing diet, whereas the other received a reduced diet. Emerging adult females were then provided an infectious blood meal. We assessed mosquito longevity, parasite development rate and prevalence of infectious mosquitoes over time. Reduced larval food led to increased adult mortality and caused a delay in parasite development and a slowing in the rate at which parasites invaded the mosquito salivary glands, extending the time it took for mosquitoes to become infectious. Together, these effects increased transmission potential of mosquitoes in the high food regime by 260–330%. Such effects have not, to our knowledge, been shown previously for human malaria and highlight the importance of improving knowledge of larval ecology to better understand vector-borne disease transmission dynamics.

## 1. Introduction

The larvae of many insects that vector human disease exhibit very different ecologies from the adult vectors ultimately responsible for disease transmission. For example, while adult vectors often live in and around domestic dwellings, larval sandflies (vectors of leishmaniasis) inhabit subterranean habitats, such as rodent burrows, and larval mosquitoes (vectors of numerous diseases including malaria, filariasis, dengue, chikungunya and Zika virus) occupy aquatic habitats ranging from freshwater streams to stagnant water in drainage ditches or man-made containers [1]. For many holometabolous insects, conditions experienced during the larval stages can carry over to influence adult life-history traits [2–4]. However, the majority of vector biology research tends to focus directly on the adult vectors, and the potential for larval ecology to have indirect influence on disease transmission is relatively less well studied.

The most extensive research exploring the effects of larval condition on the vectorial capacity of adult mosquitoes derives from *Aedes*–arbovirus systems [5–11]. For example, in adult female *Aedes albopictus*, low larval rearing temperatures decrease dissemination of dengue virus, but increase susceptibility to chikungunya virus, in comparison with adults from warmer larval environments [5,8]. *Aedes albopictus* larvae that are competitively stressed, either intraspecifically (high larval density) or interspecifically (with *Aedes aegypti*) are more likely to become infected with Sindbis virus, whereas *Ae. aegypti* exhibits equivalent susceptibility, regardless of density or competitive species [7]. Additionally, reduced larval food quantity in *Ae. aegypti* has been shown to influence the interaction between humoral and cellular branches of the immune system in adult mosquitoes, which could also directly affect vector competence for a variety of pathogens [6].

A more limited number of studies in *Anopheles* spp., show similar effects of larval environment on life-history traits and vector competence [12–19]. In *Anopheles gambiae,* high larval densities and low larval food quantity hindered first mating success of emerging adult males [18]. In areas where larval competition between *An. gambiae* and *Anopheles arabiensis* occurs, it has been demonstrated that time to pupation is reduced for *An. gambiae* and extended for *An. arabiensis* compared with larval pools containing only one species [14]. The nature of local soils (organic carbon, nitrogen, and microbial content) has also been shown to influence larval development of *An. gambiae* and subsequent susceptibility of adult mosquitoes to *Plasmodium falciparum* infection [15,16]. We recently demonstrated that a reduction in larval mosquito food quantity significantly decreases vectorial capacity of *Anopheles stephensi* infected with a rodent malaria (*Plasmodium yoelii*). We noted significant effects of larval food quantity on adult life-history traits important to transmission, such as survival, length of gonotrophic cycle, and number of emerging adults; however, we observed no effect of nutrition on parasite prevalence [17].

In spite of this research, the role of larval ecology remains poorly integrated into understanding of vector-borne disease transmission, and for human malaria in particular, few studies have explored the net effect of larval condition on both adult and parasite traits that combine to determine ultimate transmission potential [17,19,20]. In this study, we investigate how differences in the quantity of food available to larval *An. stephensi* mosquitoes affect the capacity of adult mosquitoes to vector human malaria, *P. falciparum*. We found that reducing larval food quantity reduced adult longevity, increased the parasite extrinsic incubation period (EIP) and extended the time it took for mosquitoes to become infectious. Such effects have not, to our knowledge, been reported previously for human malaria but, given the variability in larval habitat quality in nature [21–25], could play a substantial role in transmission dynamics.

## 2. Material and methods

### (a) Mosquito rearing and experimental design

*Anopheles stephensi* larvae were collected from our laboratory colony at The Pennsylvania State University (this colony was initiated in April 2014 with eggs from a longstanding colony maintained at Walter Reed Army Institute of Research, Silver Spring, MD, USA). Newly hatched (less than 24 h old) first-instar larvae were transferred to plastic trays (36 × 20 × 13 cm) containing 1 l of distilled water at initial densities of 400 larvae per tray. Larvae were maintained on Tetrafin^®^ fish food under standard insectary conditions (26°C ± 0.5°C, 80% humidity, and a 12 L : 12 D photoperiod).

From our collected first-instar larvae, we generated two colonies: (i) a ‘high’ food colony, receiving 0.6 mg of food per individual per day, which is consistent with our standard colony maintenance diet; and (ii) a ‘low’ food colony, receiving 0.2 mg of food per individual per day. The specific food treatments were chosen based on a series of pilot studies, with the goal of generating a ‘low food’ regime that still allowed for a colony that would produce sufficient numbers of emerging adult mosquitoes to perform large-scale experimental infections (electronic supplementary material, figure S1). We maintained a constant concentration of food in each tray by filtering and replacing water daily before feeding. Each group consisted of 30 trays, with an initial population of 12 000 larvae in each colony.

To ensure that mosquitoes received an infectious blood meal at the same adult age, first-instar larvae from the low food treatment group were collected 3 days earlier than first-instar larvae from the high food treatment group (electronic supplementary material, figure S1). This was to adjust for the slower developmental time owing to low food availability, and to allow for an age-matched comparison of the groups at the same time points post-infection. Although the mosquitoes from the low food colony were chronologically older, because of the additional time spent in larval development, the adult females from each colony were of equal post-emergence age at the time of infection.

In each colony, pupae from each larval tray were distributed evenly across 10 mesh emergence cages (17.5 × 17.5 × 17.5 cm, BugDorm^®^, Taichung, Taiwan). Upon adult emergence, each cage was provided with cotton balls moistened with 10% glucose, replaced daily. Early emerging females (those emerged more than 5 days pre-infectious blood meal) in each cage were removed. Three to 5 days following peak female emergence, adult females from each emergence cage were evenly distributed across 8–10 cardboard cups (475 ml) for each colony, totalling 150 females per cup (electronic supplementary material, figure S2). Each cup was provided a blood meal of human blood infected with *P. falciparum*. Dead mosquitoes were counted daily in each cup, and subsamples of live mosquitoes were removed (details below) to assess the proportion of infectious mosquitoes. Owing to logistical constraints of maintaining tray-to-cage-to-cup replication within an experiment, we replicated the experiment twice through time (experimental block 1: eight cups per treatment; block 2: 10 cups per treatment; electronic supplementary material, figure S3).

As our experimental females are drawn from a large colony pool that has been mixed on two separate occasions from pupation to infection, we included wing length as a feedback signal to ensure our colonies were producing females that were significantly different between groups. During each experimental block, we removed 10 females from each emergence cage for both food groups at the same time as the allocation of females to infectious cups for blood meals. Wing lengths were measured to the nearest micrometre using CellSens imaging software (Olympus). Wing lengths were significantly different between food groups in both blocks (electronic supplementary material, figure S4), demonstrating that our colonies were indeed producing individuals of differing condition.

### (b) *Plasmodium falciparum* culture and infection

*In vitro* cultures of *P. falciparum* strain NF54 (wild-type, Center for Infectious Disease Research, Seattle, WA, USA) were maintained in RPMI 1640 medium (25 mM HEPES, 2 mM l-glutamine), supplemented with 50 µM hypoxanthine and 10% human A+ serum (Valley Biomedical, Winchester, VA, USA). Culture was maintained in an atmosphere of 5% CO_2_, 5% O_2_ and 90% N_2_. Parasite cells were then subcultured into O+ human erythrocytes (Valley Biomedical). Gametocyte initiation occurred at 5% haematocrit and 0.8–1.0% mixed-stage parasitaemia. The culture was maintained for 17 days, and parasite cells were washed and provided fresh media daily.

Prior to the administration of the infectious blood meal, females were starved for 18 h (imbibing only water) to ensure a maximum proportion of blood feeding. On the day the infectious feed was administered to mosquitoes, gametocyte cultures (approx. 8% gametocytaemia for each experimental block) were briefly centrifuged, and the supernatant was removed and discarded. Pelleted erythrocytes were diluted to 40% haematocrit using fresh A+ human serum and O+ human erythrocytes. The mixture was pipetted into glass bell jars fixed with a Parafilm membrane and connected by plastic tubing with continuously flowing water heated to 37°C. Each bell jar was filled with 2 ml of blood culture. Mosquitoes were given 20 min to fully engorge, after which the bell jars were removed, as the parasites in culture are no longer viable. In each colony, more than 90% of females across all cups were observed to have blood in their midguts in both experimental blocks. Following blood feeding, mosquitoes were maintained at 27°C ± 0.5°C, 85% humidity, and each cup was provided with cotton balls soaked with 10% glucose and 0.05% para-aminobenzoic acid in water, which were replaced daily.

### (c) Parasite development and adult longevity

To estimate the effects of larval nutrition on parasite development rate, we assessed the daily proportion of mosquitoes that were infectious through days 9–16 following the infectious blood meal. To do this, we aspirated 8–10 mosquitoes from each replicate cup into absolute ethanol and dissected salivary glands in 0.01 M phosphate-buffered saline. Glands were ruptured and examined under a light microscope at 400× for the presence of sporozoites. Although not discussed in this study, data were also collected on parasite establishment within the mosquito midgut. We assessed both oocyst prevalence and number of oocysts per midgut via dissections from days 5 to 10 post-infection followed by observation under a light microscope (electronic supplementary material, figure S5 and tables S1 and S2).

To quantify effects of larval food quantity on adult survivorship post-infection, we counted dead mosquitoes in each replicate cup daily. For survival analysis, mosquitoes removed for dissections each day, and those still alive at the end of the experiment (day 16) were considered censored cases. We built a series of models with food and block as covariates, using the R package *flexsurv*. We compared exponential, Weibull and Gompertz distributions for survival, as these distributions have been commonly used to describe mosquito survival under laboratory conditions [26–28]. The best-fit model, which was a Gompertz curve described by larval food treatment and experimental block (electronic supplementary material, table S3), was chosen using Akaike's information criterion (AIC).

### (d) Parasite dynamics and extrinsic incubation period

The EIP describes the time it takes for the parasite to develop in the mosquito from the initial parasite-infected blood feed through to the point at which sporozoites enter the salivary glands and the mosquito becomes infectious. The number of infectious mosquitoes in a cohort of mosquitoes is expected to increase from zero to some maximum prevalence over several days [29–31], and defining EIP at different points along this cumulative curve has been shown to alter estimates of transmission intensity [29]. Whether the shape of this cumulative distribution is affected by larval diet is unknown.

To assess the effect of larval food quantity on parasite dynamics across the course of sporogony, we estimated how larval food treatment and experimental block influenced the daily mean values for sporozoite prevalence in a nonlinear logistic regression model framework [29]. Here, the change in proportion of infectious mosquitoes over time is described by a sigmoidal curve with the given equation:
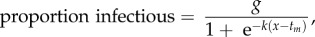
where *g* is the maximum observed sporozoite prevalence over the course of the infection, *k* is the instantaneous rate of change, *x* is time post-infection (days) and *t*_m_ is the time at which change in the proportion of infectious mosquitoes is maximal. We constructed 24 competing models to describe how the dynamics of mosquito infection varied in response to food treatment and block effects (see the electronic supplementary material, table S4 for model parameter designations and electronic supplementary material, figures S7 and S8 for individual cup dynamics in each block). The largest model had 12 parameters—i.e. treatment, block and treatment × block interaction effects on *g*, *t*_m_ and *k*, whereas our null model contained only the three parameters *g, k* and *t*_m_. We used the AIC to assess which model fitted our data best (electronic supplementary material, table S5).

### (e) Estimating transmission potential

One of the standard ways to characterize transmission potential is vectorial capacity, which describes the daily rate at which future infections arise from one infected human, given that all mosquitoes feeding on that human become infected [32]. However, vectorial capacity assumes there is a single fixed value for EIP and that there is a constant rate of daily adult mosquito survival, neither of which fit with our empirical data. Accordingly, we used an alternative measure, adapted from the work of Killeen *et al*. [33] to describe the transmission potential of a cohort of mosquitoes (see also [34] for analogous methods). In this framework, we assessed the number of mosquitoes that are both alive and infectious at any given time by overlaying our survival curves with our model fits for change in mosquito infectiousness over time. We extended our survival estimates to the point where just 1% of mosquitoes were predicted to be alive, as the Gompertz survival curves never reach zero. We follow the standard assumption that once sporozoites have invaded the salivary glands, a mosquito remains infectious for life [32,35,36].

This approach enables us to capture the interacting dynamics of sporogony and survival. The product of these two proportions (the area under the curve) represents the cumulative number of ‘infectious mosquito days’. This value is then multiplied by the daily biting rate to provide an estimate of the probable number of infectious bites transmitted by a cohort of mosquitoes, assuming all blood meals are taken on humans (analogous to force of infection for a given mosquito cohort). Here, we illustrate our model results by assuming an initial cohort of 100 mosquitoes for each treatment group. For our estimate of daily biting rate, we followed convention by taking the reciprocal of the gonotrophic cycle length [29,35,36] as *An. stephensi* have not been observed to take multiple blood meals per gonotrophic cycle in the field. We used empirically observed values of gonotrophic cycle length from a pilot experiment conducted with these same food regimes using the same stock colony (see the electronic supplementary material, figure S4 and also [17] for similar data).

## 3. Results

### (a) Post-infectious longevity

**Figure 1.**
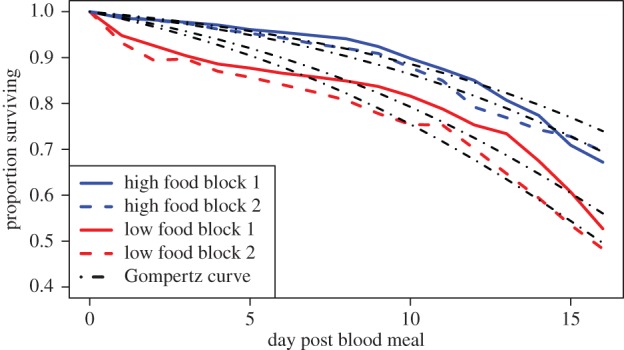
Proportion of mosquitoes surviving over time, with the predicted Gompertz distribution overlaid on raw survival data. Parameter estimates: shape (*β*) = 0.01184; high food block 1 rate (*α*) = 0.00632; low food block 1 rate (*α*) = 0.01214, high food block 2 rate (*α*) = 0.00765, low food block 2 rate (*α*) = 0.01470. The *x*-axis begins at 0.4 for ease of visualization.

Larval food treatment significantly affected daily survival of blood-fed adult females (figure 1). Median survival time predicted by our Gompertz function for females from the high food groups was 22.3 days (95% confidence interval (CI) = 21.2–23.5 days) and 20.8 days (95% CI = 19.9–21.8 days) for blocks 1 and 2, respectively, and 17.3 days (95% CI = 16.6–18.1 days) and 15.9 days (95% CI = 15.3–16.6 days) for females from the low food groups in blocks 1 and 2, respectively.

### (b) Effects of treatment on the extrinsic incubation period and parasite dynamics

**Figure 2.**
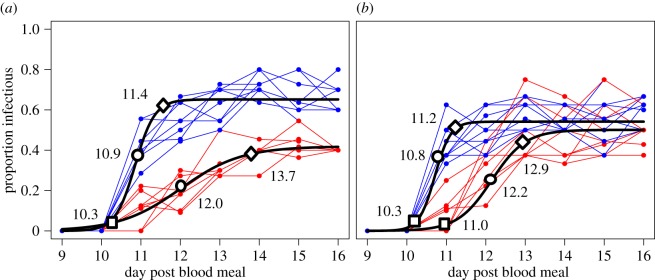
Raw data for sporozoite prevalence over time for (*a*) block 1 and (*b*) block 2; (coloured points, blue = high food, red = low food) fitted with our best-fit binary logistic regression model (black line). Lines connecting points represent separate experimental cups within each treatment. To illustrate the change in differences between groups through time, shapes along the black line represent time to 10% (open squares), 50% (open circles) and 90% (open diamonds) of maximum infectiousness. The numbers aside each shape are the corresponding days to reach that point.

Our best-fit logistic regression model suggested important effects of larval food treatment on the cumulative distribution of infectious mosquitoes over time (figure 2 and table 1). This model was significantly better than the fully reduced model containing only the original terms *g*, *k* and *t*_m_ (electronic supplementary material, table S5).

**Table 1.** Estimates for best-fit logistic model parameter values describing the kinetics of parasite development within the adult mosquitoes for each block and treatment combination.

parameter	low food block 1	high food block 1	low food block 2	high food block 2
asymptote (*g*)	0.4189	0.6519	0.5009	0.5416
rate (*k*)	−1.2967	−4.1039	−2.3244	−5.0417
inflection (*t*_m_)	12.0064	10.8519	12.1496	10.7087

We observed an overall reduction (significant in block 1) in the maximum proportion of infectious mosquitoes (*g*) in the reduced food treatment (figure 2). We also observed differences in the rates of parasite development between treatments with higher rates of change (*k*) in the high food treatment compared with the low food treatment, irrespective of asymptotic differences in maximum prevalence (figure 2 and table 1).

As shown in figure 2, the logistic model indicated that the initial release of sporozoites within the population (which we characterize as time to 10% of maximum infectiousness) occurred at 10.3 days post-blood meal in the high food treatment in both blocks 1 and 2. For the low food treatment, 10% infectiousness also occurred at day 10.3 and day 11 in blocks 1 and 2, respectively. As the infection proliferated within the population, the differences in parasite development between the two treatment groups increased. The time to 50% of maximum infectiousness (the median incubation period, indicated by the inflection point) occurred at 10.9 and 10.7 days for blocks 1 and 2 in the high food treatments, and 12.1 days for the low food treatments in both experimental blocks. The time to 90% infection (i.e. approaching the maximum prevalence, *g*) was 11.4 and 11.2 days in blocks 1 and 2 for the high food treatment but 13.7 and 12.9 days for the low food treatment.

### (c) Estimating treatment effects on transmission potential

**Figure 3.**
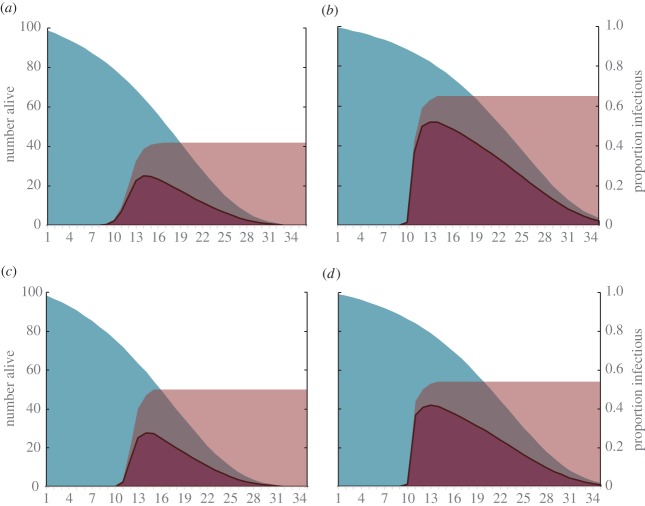
Area curves for rates of survival (blue) and infection (pink) for each block and treatment combination: (*a*) low food, block 1; (*b*) high food block 1; (*c*) low food block 2; and (*d*) high food block 2. Purple areas represent the product of the two curves (i.e. the number of mosquitoes alive and infectious).

Using our metric of transmission potential, we see a clear difference in the number of cumulative infectious mosquito days between the two larval food groups (area under the curve indicated in figure 3). Assuming an initial cohort of 100 mosquitoes, the resulting values represent the probable number of infectious bites transmitted for that cohort until only 1% of the population is remaining. After multiplying the number of cumulative infectious mosquito days by daily biting rate, we observe transmission potential values for the high food group of 247 and 186 infectious bites for blocks 1 and 2, respectively. For the low food groups, we observe values of 74 and 71 infectious bites for blocks 1 and 2, respectively. Overall, transmission potential of mosquitoes in the high food regime was increased by 260–330% relative to the low food group (table 2).

**Table 2.** Calculations of transmission potential for each food treatment and block combination, and the relative increase in transmission potential from low food to high food.

treatment	block	cumulative infectious mosquito days	biting rate	transmission potential	increase (%)
high food	1	718.32	0.344	247.102	330
low food	1	260.79	0.283	73.804	
high food	2	539.59	0.344	185.619	260
low food	2	251.70	0.283	71.231	

## 4. Discussion

Numerous studies have demonstrated that larval conditions in holometabolous insects can carry over to affect adult life-history traits. The aim of the current experiments was to test the hypothesis that differences in larval food quantities can affect the potential of adult *An. stephensi* mosquitoes to vector the human malaria parasite *P. falciparum.* Our results show that changes in larval food can lead to substantial changes in the force of infection of a cohort of mosquitoes (table 2).

One effect of variation in larval food was an impact on daily adult survival. This effect is not surprising given that adult survival has been shown to be directly correlated to larval nutritional status in many mosquito species [11,13,17,36,37], including uninfected *Anopheles* species [37,38], and in *Anopheles* adults infected with rodent malaria species [13,17].

Perhaps less expected was the clear influence of larval food quantity on parasite development within adult mosquitoes. Although we did not note a significant difference in parasite prevalence between food treatments in our second experimental block, the effects of larval nutrition on the inflection point and instantaneous rate of change of our model curves describing parasite growth kinetics were consistent between blocks. This result suggests a robust effect of larval food on parasite development. Further, the effect of larval diet on the time taken to reach maximum infectiousness suggests an influence of larval nutrition during energetically demanding stages of oocyst maturation.

There currently is limited knowledge on what nutrients *Plasmodium* parasites use from their mosquito hosts. *Plasmodium*-infected mosquitoes have been shown to contain significantly lower amounts of amino acids, glucose and sterols compared with non-infected, blood-fed conspecifics, suggesting that parasite development may influence the composition and quantity of mosquito reserves [39–41]. *Plasmodium* spp. are also known to scavenge fatty acids from their mosquito and vertebrate hosts, despite their ability to synthesize fatty acids de novo [40,42], and laboratory observations of *Aedes* and *Anopheles* spp. infected with avian and rodent malaria, respectively, show that the mosquito lipoprotein lipophorin is essential for the development and maturation of oocysts [43–45]. Finally, numerous studies indicate that poor larval diet drastically reduces the relative amount of proteins, glycogen stores and total lipids per microgram of adult body mass in *Anopheles* and *Aedes* species [13,46–50]. Hence, it is possible that differences in larval food quantity result in variation in levels of compounds essential to oocyst maturation in adult female mosquitoes.

Research in other insect-parasite systems indicates that host nutritional status can affect parasite development. For example, in bumblebees infected with the trypanosome *Crithidia bombi*, starving adult bees of pollen resulted in significantly decreased parasite intensity. Additionally, the life cycle of *C. bombi* is normally synchronized in healthy bees, but in starved bees, parasite populations were observed to comprise a mix of parasite life cycle stages [51,52]. Similar dynamics have been observed in triatomine bugs infected with *Trypanosoma cruzi* (the causative agent of Chagas' disease in humans), with evidence that quality and quantity of blood meals can directly influence parasite development [53,54]. These findings contrast with a recent study on *Ae. aegypti*, which showed no impact of larval rearing condition on the EIP of dengue virus in spite of clear effects on life-history traits such as adult longevity [55].

Our approach to describing overall transmission potential (force of infection for a given mosquito cohort) better captures the dynamic interaction of sporogony and survival than conventional static metrics such as vectorial capacity [32,35,56,57]. All derivations of the vectorial capacity equation assume a single value for EIP for a particular mosquito population or environment (typically the first time point that mosquitoes become infectious). However, our data clearly show sporogony to follow a distribution that cannot be simply represented by a single time point (see [34] for analogous patterns in dissemination of dengue virus in *Aedes aegypti*). Additionally, mosquito survival is typically characterized using an exponential function that assumes a constant rate of survival per day. Our data show mortality to follow an age-dependent Gompertz distribution, a result consistent with numerous studies on survival of caged *Anopheles*, *Culex* and *Aedes* species [26–28,58,59]. We note, however, that we did not follow adult survival until all mosquitoes were dead and so cannot confirm the complete shape of the survival function empirically. Additionally, mortality in the field is likely to be dependent on many factors, including temperature, rainfall, humidity, predation and habitat (e.g. rural or urban) [21–25,59–62].

We acknowledge that our results derive from laboratory-adapted strains of mosquito and parasite. In the field, we might expect local strains to vary in terms of key life-history traits [63]. The use of the standard mass-rearing larval diet of fish food could also influence parasite dynamics. However, what constitutes an ‘optimal’ diet in the field is unclear as larval habitats are diverse [21–25,64,65]. Furthermore, the effects of varying nutrition ought to be fundamental: it has been demonstrated in multiple studies that varying nutrition will affect size, longevity, fecundity, gonotrophic cycle and other life-history traits [13,17,46,66–68], so there is little reason to assume the qualitative alterations in traits we observe are an artefact of the laboratory system alone. Consistent with this argument, seasonal quality of larval habitats has been shown to influence adult body size in field populations of *Anopheles* species [68,69]*.* Natural variation in body size of field-collected *An. gambiae* has also been shown to influence probability of eventual sporozoite infection [70]. The same is true for other disease vectors, with the frequency of dengue infection increasing with female body size in *Ae. aegypti* [71].

Recent years have seen a substantial body of research exploring potential effects of environmental drivers, such as temperature, on malaria transmission [29,72–77]. Our results demonstrate that variation in larval nutrition can impact transmission potential with equivalent effect size to typical variation in environmental temperature (including predicted effects of climate change), yet the role of larval ecology in both current and future disease dynamics has been largely overlooked to date. That the effects we observe are substantial provides important motivation for future research to examine these processes with field strains under field conditions. Quantifying how larval traits feed through to impact malaria transmission could improve understanding of disease dynamics and inform the development of improved vector control strategies.

## Supplementary Material

Shapiro et al. 2016 ESM

## Data accessibility

All relevant data and R scripts are available at http://dx.doi.org/10.5061/dryad.d1b7h.

## References

[1] WHO. 2014 *A global brief on vector borne diseases*. Geneva, Switzerland: World Health Organization Press.

[2] De Block M, Stoks R 2008 Short-term larval food stress and associated compensatory growth reduce adult immune function in a damselfly. Ecol. Entomol. 33, 796–801. (10.1111/j.1365-2311.2008.01024.x)

[3] Campero M, De Block M, Ollevier F, Stoks R 2008 Metamorphosis offsets the link between larval stress, adult asymmetry and individual quality. Funct. Ecol. 22, 271–277. (10.1111/j.1365-2435.2007.01381.x)

[4] Perdikis C 2008 Effect of larval crowding on the life history traits of *Sesamia nonagrioides* (Lepidoptera: Noctuidae). Eur. J. Entomol. 5759, 625–630.

[5] Westbrook CJ, Reiskind MH, Pesko KN, Greene KE, Lounibos LP 2010 Larval environmental temperature and the susceptibility of *Aedes albopictus* Skuse (Diptera: Culicidae) to chikungunya virus. Vector-borne Zoonotic Dis. 10, 241–247. (10.1089/vbz.2009.0035) 19725768 PMC2883477

[6] Telang A, Qayum AA, Parker A, Sacchetta BR, Byrnes GR 2012 Larval nutritional stress affects vector immune traits in adult yellow fever mosquito *Aedes aegypti* (*Stegomyia aegypti*). Med. Vet. Entomol. 26, 271–281. (10.1111/j.1365-2915.2011.00993.x) 22112201

[7] Alto BW, Lounibos LP, Higgs S, Juliano SA 2005 Larval competition differentially affects arbovirus infection in *Aedes* mosquitoes. Ecology 86, 3279–3288. (10.1890/05-0209) 19096729 PMC2605070

[8] Alto BW, Reiskind MH, Lounibos LP 2008 Size alters susceptibility of vectors to dengue virus infection and dissemination. Am. J. Trop. Med. Hyg. 79, 688–695. 18981505 PMC2630770

[9] Alto BW, Bettinardi D 2013 Temperature and dengue virus infection in mosquitoes: independent effects on the immature and adult stages. Am. J. Trop. Med. Hyg. 88, 497–505. (10.4269/ajtmh.12-0421) 23382163 PMC3592531

[10] Joy TK, Arik AJ, Corby-Harris V, Johnson AA, Riehle MA 2010 The impact of larval and adult dietary restriction on lifespan, reproduction and growth in the mosquito *Aedes aegypti*. Exp. Gerontol. 45, 685–690. (10.1016/j.exger.2010.04.009) 20451597 PMC4181608

[11] Muturi EJ, Blackshear M, Montgomery A 2012 Temperature and density-dependent effects of larval environment on *Aedes aegypti* competence for an alphavirus. J. Vector Ecol. 37, 154–161. (10.1111/j.1948-7134.2012.00212.x) 22548549

[12] Lyimo EO, Takken W, Koella JC 1992 Effect of rearing temperature and larval density on larval survival, age at pupation and adult size of *Anopheles gambiae*. Entomol. Exp. Appl. 63, 265–271. (10.1111/j.1570-7458.1992.tb01583.x)

[13] Takken W, Smallegange RC, Vigneau AJ, Johnston V, Brown M, Mordue-Luntz A, Billingsley PF 2013 Larval nutrition differentially affects adult fitness and *Plasmodium* development in the malaria vectors *Anopheles gambiae* and *Anopheles stephensi*. Parasit. Vectors 6, 345 (10.1186/1756-3305-6-345) 24326030 PMC4029273

[14] Paaijmans KP, Huijben S, Githeko AK, Takken W 2009 Competitive interactions between larvae of the malaria mosquitoes *Anopheles arabiensis* and *Anopheles gambiae* under semi-field conditions in western Kenya. Acta Trop. 109, 124–130. (10.1016/j.actatropica.2008.07.010) 18760989

[15] Pfaehler O, Oulo DO, Gouagna LC, Githure J, Guerin PM 2006 Influence of soil quality in the larval habitat on development of *Anopheles gambiae* Giles. J. Vector Ecol. 31, 400–405. (10.3376/1081-1710(2006)31) 17249359

[16] Okech BA et al. 2007 Larval habitats of *Anopheles gambiae* s.s. (Diptera: Culicidae) influences vector competence to *Plasmodium falciparum* parasites. Malar. J. 6, 50 (10.1186/1475-2875-6-50) 17470293 PMC1868750

[17] Moller-Jacobs LL, Murdock CC, Thomas MB 2014 Capacity of mosquitoes to transmit malaria depends on larval environment. Parasit. Vectors 7, 593 (10.1186/s13071-014-0593-4) 25496502 PMC4273441

[18] Ng'habi KR, John B, Nkwengulila G, Knols BGJ, Killeen GF, Ferguson HM 2005 Effect of larval crowding on mating competitiveness of *Anopheles gambiae* mosquitoes. Malar. J. 4, 49 (10.1186/1475-2875-4-49) 16197541 PMC1260028

[19] Roux O et al. 2015 Evidence for carry-over effects of predator exposure on pathogen transmission potential. Proc. R. Soc. B 282, 20152430 (10.1098/rspb.2015.2430) PMC4707764 26674956

[20] Vantaux A, Ouattarra I, Lefèvre T, Dabiré KR 2016 Effects of larvicidal and larval nutritional stresses on *Anopheles gambiae* development, survival and competence for *Plasmodium falciparum*. Parasit. Vectors 9, 226 (10.1186/s13071-016-1514-5) 27107591 PMC4842262

[21] Mehravaran A et al. 2012 Ecology of *Anopheles stephensi* in a malarious area, southeast of Iran. Acta Med. Iran. 50, 61–65. 22267381

[22] Sinka ME et al. 2012 A global map of dominant malaria vectors. Parasit. Vectors 5, 69 (10.1186/1756-3305-5-69) 22475528 PMC3349467

[23] Ndenga BA, Simbauni JA, Mbugi JP, Githeko AK, Fillinger U 2011 Productivity of malaria vectors from different habitat types in the western Kenya Highlands. PLoS ONE 6, e19473 (10.1371/journal.pone.0019473) 21559301 PMC3085476

[24] Pemola Devi N, Jauhari RK 2007 Mosquito species associated within some Western Himalayan phytogeographic zones in the Garhwal region of India. J. Insect Sci. 7, 1–10. (10.1673/031.007.3201) PMC2999433 20233101

[25] Ageep TB et al. 2009 Spatial and temporal distribution of the malaria mosquito *Anopheles arabiensis* in northern Sudan: influence of environmental factors and implications for vector control. Malar. J. 8, 123 (10.1186/1475-2875-8-123) 19500425 PMC2698915

[26] Christiansen-Jucht C, Erguler K, Shek C, Basáñez M-G, Parham P 2015 Modelling *Anopheles gambiae* s.s. population dynamics with temperature- and age-dependent survival. Int. J. Environ. Res. Public Health 12, 5975–6005. (10.3390/ijerph120605975) 26030468 PMC4483682

[27] Brady OJ et al. 2013 Modelling adult *Aedes aegypti* and *Aedes albopictus* survival at different temperatures in laboratory and field settings. Parasit. Vectors 6, 351 (10.1186/1756-3305-6-351) 24330720 PMC3867219

[28] Styer LM, Carey JR, Wang JL, Scott TW 2007 Mosquitoes do senesce: departure from the paradigm of constant mortality. Am. J. Trop. Med. Hyg. 76, 111–117. (10.1016/j.biotechadv.2011.08.021) 17255238 PMC2408870

[29] Paaijmans KP, Blanford S, Chan BHK, Thomas MB 2012 Warmer temperatures reduce the vectorial capacity of malaria mosquitoes. Biol. Lett. 8, 465–468. (10.1098/rsbl.2011.1075) 22188673 PMC3367745

[30] Vaughan JA 2007 Population dynamics of *Plasmodium* sporogony. Trends Parasitol. 23, 63–70. (10.1016/j.pt.2006.12.009) 17188574

[31] Vaughan JA, Noden BH, Beier JC 1992 Population dynamics of *Plasmodium falciparum* sporogony in laboratory-infected *Anopheles gambiae*. J. Parasitol. 78, 716–724. (10.2307/3283550) 1635032

[32] Garrett-Jones C, Shidrawi GR 1969 Malaria vectorial capacity of a population of *Anopheles gambiae*: an exercise in epidemiological entomology. Bull. World Health Organ. 40, 531–545. 5306719 PMC2556109

[33] Killeen GF, McKenzie FE, Foy BD, Schieffelin C, Billingsley PF, Beier JC 2000 A simplified model for predicting malaria entomologic inoculation rates based on entomologic and parasitologic parameters relevant to control. Am. J. Trop. Med. Hyg. 62, 535–544. 11289661 10.4269/ajtmh.2000.62.535 PMC2483339

[34] Christofferson RC, Mores CN 2011 Estimating the magnitude and direction of altered arbovirus transmission due to viral phenotype. PLoS ONE 6, e16298 (10.1371/journal.pone.0016298) 21298018 PMC3029343

[35] Macdonald G 1957 The epidemiology and control of malaria. London, UK: Oxford University Press.

[36] Smith DL, McKenzie FE 2004 Statics and dynamics of malaria infection in *Anopheles* mosquitoes. Malar. J. 3, 13 (10.1186/1475-2875-3-13) 15180900 PMC449722

[37] Afrane YA, Zhou G, Lawson BW, Githeko AK, Yan G 2007 Life-table analysis of *Anopheles arabiensis* in Western Kenya Highlands: effects of land covers on larval and adult survivorship. Am. J. Trop. Med. Hyg. 77, 660–666. 17978067

[38] Ameneshewa B, Service MW 1996 The relationship between female body size and survival rate of the malaria vector *Anopheles arabiensis* in Ethiopia. Med. Vet. Entomol. 10, 170–172. (10.1111/j.1365-2915.1996.tb00724.x) 8744710

[39] Mack SR, Samuels S, Vanderberg JP 1979 Hemolymph of *Anopheles stephensi* from uninfected and *Plasmodium berghei*-infected mosquitoes. 2. Free amino acids. J. Parasitol. 65, 130–136. (10.2307/3280217) 376812

[40] Ginger ML 2006 Niche metabolism in parasitic protozoa. Phil. Trans. R. Soc. Lond. B 361, 101–118. (10.1098/rstb.2005.1756) 16553311 PMC1626543

[41] Nyasembe VO, Teal PEA, Sawa P, Tumlinson JH, Borgemeister C, Torto B 2014 *Plasmodium falciparum* infection increases *Anopheles gambiae* attraction to nectar sources and sugar uptake. Curr. Biol. 24, 217–221. (10.1016/j.cub.2013.12.022) 24412210 PMC3935215

[42] van Schaijk BCL et al. 2014 Type II fatty acid biosynthesis is essential for *Plasmodium falciparum* sporozoite development in the midgut of *Anopheles* mosquitoes. Eukaryot. Cell 13, 550–559. (10.1128/EC.00264-13) 24297444 PMC4060470

[43] Atella GC, Bittencourt-Cunha PR, Nunes RD, Shahabuddin M, Silva-Neto MAC 2009 The major insect lipoprotein is a lipid source to mosquito stages of malaria parasite. Acta Trop. 109, 159–162. (10.1016/j.actatropica.2008.10.004) 19013123

[44] Cheon HM, Sang WS, Bian G, Park JH, Raikhel AS 2006 Regulation of lipid metabolism genes, lipid carrier protein lipophorin, and its receptor during immune challenge in the mosquito *Aedes aegypti*. J. Biol. Chem. 281, 8426–8435. (10.1074/jbc.M510957200) 16449228

[45] Vlachou D, Kafatos FC 2005 The complex interplay between mosquito positive and negative regulators of *Plasmodium* development. Curr. Opin. Microbiol. 8, 415–421. (10.1016/j.mib.2005.06.013) 15996894

[46] Telang A, Frame L, Brown MR 2007 Larval feeding duration affects ecdysteroid levels and nutritional reserves regulating pupal commitment in the yellow fever mosquito *Aedes aegypti* (Diptera: Culicidae). J. Exp. Biol. 210, 854–864. (10.1242/jeb.02715) 17297145

[47] Briegel H 1990 Metabolic relationship between female body size, reserves, and fecundity of *Aedes aegypti*. J. Insect Physiol. 36, 165–172. (10.1016/0022-1910(90)90118-Y)

[48] Timmermann SE, Briegel H 1999 Larval growth and biosynthesis of reserves in mosquitoes. J. Insect Physiol. 45, 461–470. (10.1016/S0022-1910(98)00147-4) 12770329

[49] Hood-Nowotny R et al. 2012 An analysis of diet quality, how it controls fatty acid profiles, isotope signatures and stoichiometry in the malaria mosquito *Anopheles arabiensis*. PLoS ONE 7, e45222 (10.1371/journal.pone.0045222) 23133509 PMC3484992

[50] Komínková D, Rejmánková E, Grieco J, Achee N 2012 Fatty acids in anopheline mosquito larvae and their habitats. J. Vector Ecol. 37, 382–395. (10.1111/j.1948-7134.2012.00242.x) 23181863

[51] Logan A, Ruiz-González MX, Brown MJF 2005 The impact of host starvation on parasite development and population dynamics in an intestinal trypanosome parasite of bumble bees. Parasitology 130, 637–642. (10.1017/S0031182005007304) 15977900

[52] Brown MJF, Loosli R 2000 Condition-dependent expression of virulence in a trypanosome infecting bumblebee. Oikos 91, 421–427. (10.1034/j.1600-0706.2000.910302.x)

[53] Kollien AH, Schaub GA 2000 The development of *Trypanosoma cruzi* in Triatominae. Parasitol. Today 16, 381–387. (10.1016/S0169-4758(00)01724-5) 10951597

[54] Garcia ES, Azambuja P 1991 Development and interactions of *Trypanosoma cruzi* within the insect vector. Parasitol. Today 7, 240–244. (10.1016/0169-4758(91)90237-I) 15463507

[55] Bara J, Rapti Z, Cáceres CE, Muturi EJ 2015 Effect of larval competition on extrinsic incubation period and vectorial capacity of *Aedes albopictus* for dengue virus. PLoS ONE 10, 1–18. (10.1371/journal.pone.0126703) PMC4423876 25951173

[56] Massad E, Coutinho FAB 2012 Vectorial capacity, basic reproduction number, force of infection and all that: formal notation to complete and adjust their classical concepts and equations. Mem. Inst. Oswaldo Cruz 107, 564–567. (10.1590/S0074-02762012000400022) 22666873

[57] Dye C 1986 Vectorial capacity: must we measure all its components? Parasitol. Today 2, 203–209. (10.1016/0169-4758(86)90082-7) 15462840

[58] Clements A, Paterson GD 1981 The analysis of mortality and survival rates in wild populations of mosquitoes. J. Appl. Ecol. 18, 373–399. (10.2307/2402401)

[59] Parham PE, Pople D, Christiansen-Jucht C, Lindsay S, Hinsley W, Michael E 2012 Modeling the role of environmental variables on the population dynamics of the malaria vector *Anopheles gambiae* sensu stricto. Malar. J. 11, 271 (10.1186/1475-2875-11-271) 22877154 PMC3496602

[60] Roy DN 1931 Natural breeding habits of *Anopheles stephensi* as observed in Calcutta. Indian J. Med. Res. 19, 617–628.

[61] Louca V, Lucas M, Green C, Majambere S, Fillinger U, Lindsay S 2008 Role of fish as predators of mosquito larvae on the floodplain of the Gambia River. J. Med. Entomol. 15, 1203–1214. (10.1016/j.drugalcdep.2008.02.002.A) PMC2739801 19496426

[62] Fillinger U, Lindsay SW 2011 Larval source management for malaria control in Africa: myths and reality. Malar. J. 10, 353 (10.1186/1475-2875-10-353) 22166144 PMC3273449

[63] Joy DA, Gonzalez-Ceron L, Carlton JM, Gueye A, Fay M, McCutchan TF, Su X 2008 Local adaptation and vector-mediated population structure in *Plasmodium vivax* malaria. Mol. Biol. Evol. 25, 1245–1252. (10.1093/molbev/msn073) 18385220 PMC2386084

[64] Kweka EJ et al. 2011 Evaluation of two methods of estimating larval habitat productivity in Western Kenya Highlands. Parasit. Vectors 5, 33 (10.1186/1756-3305-5-33) PMC3138440 21682875

[65] Himeidan YE et al. 2009 Habitat stability and occurrences of malaria vector larvae in Western Kenya Highlands. Malar. J. 8, 234 (10.1186/1475-2875-8-234) 19845968 PMC2771030

[66] Hancock RG, Foster WA 1997 Larval and adult nutrition effects on blood/nectar choice of *Culex nigripalpus* mosquitoes. Med. Vet. Entomol. 11, 112–122. (10.1111/j.1365-2915.1997.tb00299.x) 9226638

[67] Araújo M, Gil LHS, e-Silva A 2012 Larval food quantity affects development time, survival and adult biological traits that influence the vectorial capacity of *Anopheles darlingi* under laboratory conditions. Malar. J. 11, 261 (10.1186/1475-2875-11-261) 22856645 PMC3469369

[68] Russell TL, Lwetoijera DW, Knols BGJ, Takken W, Killeen GF, Ferguson HM 2011 Linking individual phenotype to density-dependent population growth: the influence of body size on the population dynamics of malaria vectors. Proc. R. Soc. B. 278, 3142–3151. (10.1098/rspb.2011.0153) PMC3158942 21389034

[69] Hidalgo K, Dujardin J-P, Mouline K, Dabiré RK, Renault D, Simard F 2015 Seasonal variation in wing size and shape between geographic populations of the malaria vector *Anopheles coluzzii* in Burkina Faso (West Africa). Acta Trop. 143, 79–88. (10.1016/j.actatropica.2014.12.014) 25579425

[70] Lyimo EO, Koella JC 1992 Relationship between body size of adult *Anopheles gambiae s.l*. and infection with the malaria parasite *Plasmodium falciparum*. Parasitology 104, 233–237. (10.1017/S0031182000061667) 1594289

[71] Juliano SA, Ribeiro GS, Maciel-de-Freitas R, Castro MG, Codeço C, Lourenço-de-Oliveira R, Lounibos LP 2014 She's a femme fatale: low-density larval development produces good disease vectors. Mem. Inst. Oswaldo Cruz 109, 1070–1077. (10.1590/0074-02760140455) 25591112 PMC4325623

[72] Mordecai EA et al. 2013 Optimal temperature for malaria transmission is dramatically lower than previously predicted. Ecol. Lett. 16, 22–30. (10.1111/ele.12015) 23050931

[73] Paaijmans KP, Read AF, Thomas MB 2009 Understanding the link between malaria risk and climate. Proc. Natl Acad. Sci. USA 106, 13 844–13 849. (10.1073/pnas.0903423106) PMC2720408 19666598

[74] Blanford JI, Blanford S, Crane RG, Mann ME, Paaijmans KP, Schreiber KV, Thomas MB 2013 Implications of temperature variation for malaria parasite development across Africa. Sci. Rep. 3, 1300 (10.1038/srep01300) 23419595 PMC3575117

[75] Alonso D, Bouma MJ, Pascual M 2011 Epidemic malaria and warmer temperatures in recent decades in an East African highland. Proc. R. Soc. B 278, 1661–1669. (10.1098/rspb.2010.2020) PMC3081772 21068045

[76] Parham PE, Michael E 2010 Modeling the effects of weather and climate change on malaria transmission. Environ. Health Perspect. 118, 620–626. (10.1289/ehp.0901256) 20435552 PMC2866676

[77] Murdock CC et al. 2016 Malaria transmission potential could be reduced with current and future climate change. Sci. Rep. 6, 27771 (10.1038/srep27771) 27324146 PMC4914975

