# Supplementary material for: Larval food quantity affects the capacity of adult mosquitoes to transmit human malaria
Source: Proc Biol Sci. 2016 Jul 13;283(1834):20160298. doi: 10.1098/rspb.2016.0298 (PMC4947883; doi:10.1098/rspb.2016.0298)
Supplement: Shapiro et al. 2016 ESM [file rspb20160298supp1.pdf]

Supplementary Figure S1.

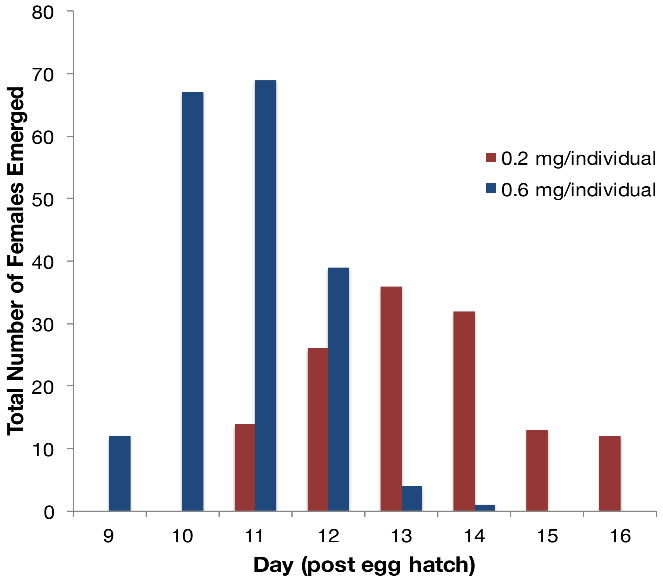

Figure S1. Distribution of female emergence over time for pilot study conducted on larvae from the Walter Reed *Anopheles stephensi* colony received in April 2014. Total number of females emerged represent all females emerged from ten cups, each initially containing 50 larvae (500 larvae per treatment). Only data from the treatment groups used in this study are shown, though we tested over a wide spectrum of food quantities. 0.2 mg/individual/day was the lowest food quantity we could administer while still observing regular female emergence. Any amount lower resulted in few larvae surviving to pupation, and not enough females to carry out an experiment of this size.

Supplementary Figure S2.

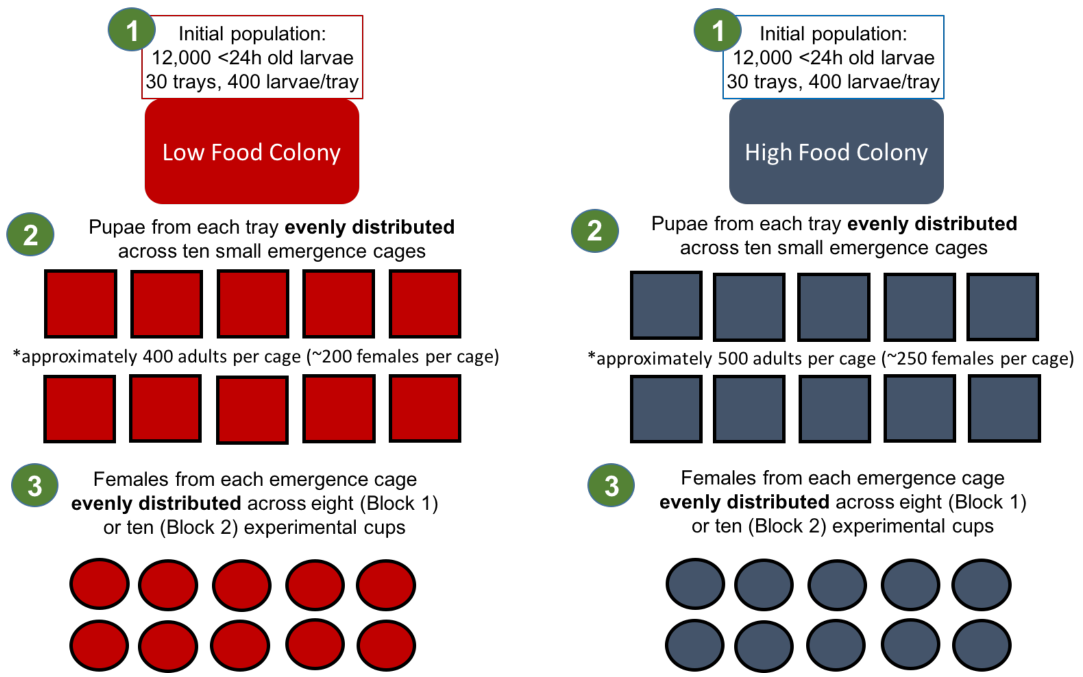

Figure S2. Schematic diagram of how mosquitoes from initial colonies were randomly allocated twice across the experiment.

Supplementary Figure S3.

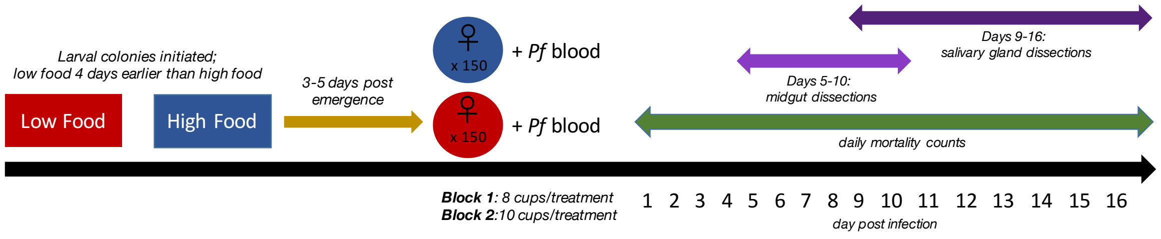

Figure S3. Schematic diagram depicting each step of the methodology used for this study and the associated sample sizes for each experimental block.

Supplementary Figure S4.

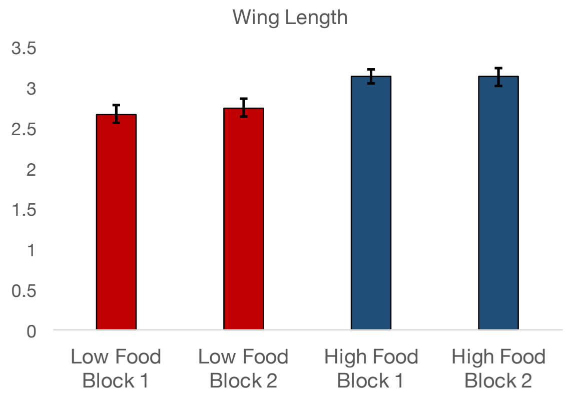

Figure S4. Mean wing length of females collected from each food group in each block. Bars represent approximately ten females from each of ten emergence cages. Error bars represent the standard deviation from the mean. Wing lengths are significantly different between food groups ($p < 0.001$ at $\alpha = 0.05$, ANOVA). Mean wing length (mm), Low Food Block 1 = 2.67 ± .106 (n = 98), Low Food Block 2 = 2.75 ± .107 (n = 90), High Food Block 1 = 3.14 ± .089 (n = 103), High Food Block 2 = 3.13 ± .106 (n = 105).

Supplementary Figure S5.

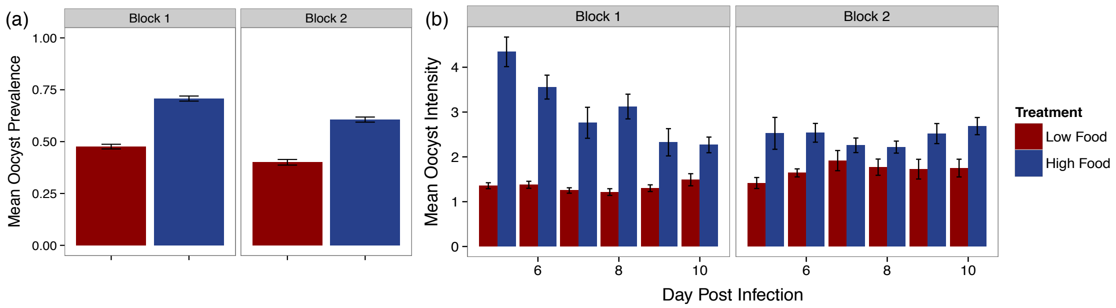

Figure S5. a. Overall mean oocyst prevalence for low food (red) and high food (blue) for each experimental block. Treatments are significantly different from each other ($p<0.001$) and blocks are significantly different from each other ($p<0.001$). Error bars represent standard error of the mean. b. Mean daily oocyst intensity for low food (red) and high food (blue) for each experimental block. Treatments are significantly different from each other ($p<0.001$) at $\alpha=0.05$.

Supplementary Figure S6.

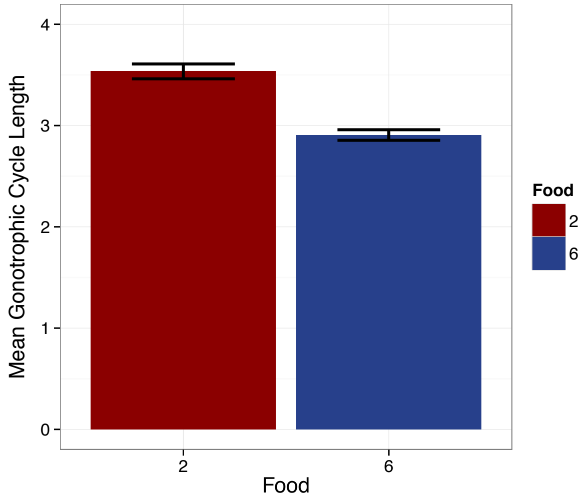

Figure S6. Observed values for gonotrophic cycle in pilot studies of the Walter Reed *Anopheles stephensi* colony. Represented here are the treatments used in this paper. Red = 0.2 mg/individual/day ("Low Food", n=86), blue = 0.6 mg/individual/day ("High Food", n=107). Mean gonotrophic cycle for Low Food = 3.53 days, biting rate a = 0.283, High Food = 2.91 days, biting rate a = .344. Error bars represent standard error of the mean. Difference between treatments is significant using a Wilcoxon rank sum test, $p<0.001$ at $\alpha=0.05$.

Supplementary Figure S7.

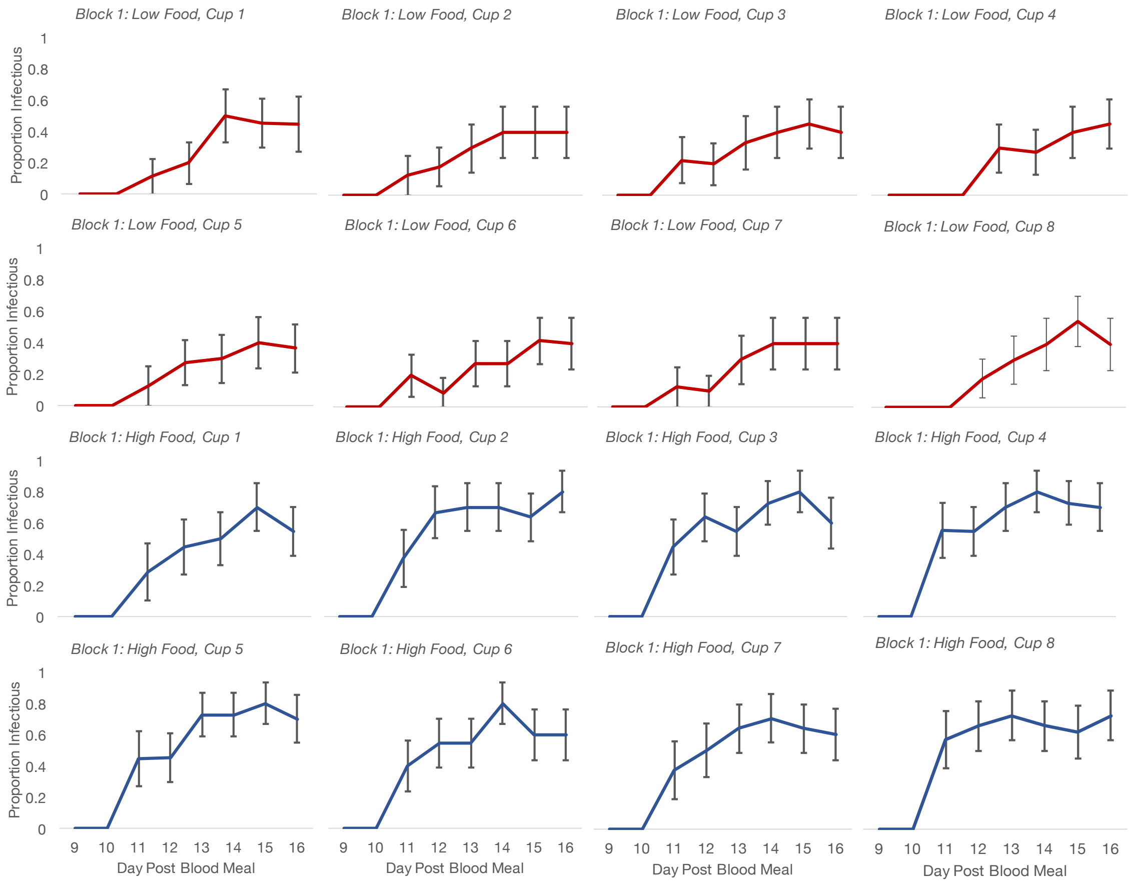

Figure S7. Dynamics of the change in proportion of infectious mosquitoes over time for each replicate cup in experimental block 1.

Supplementary Figure S8.

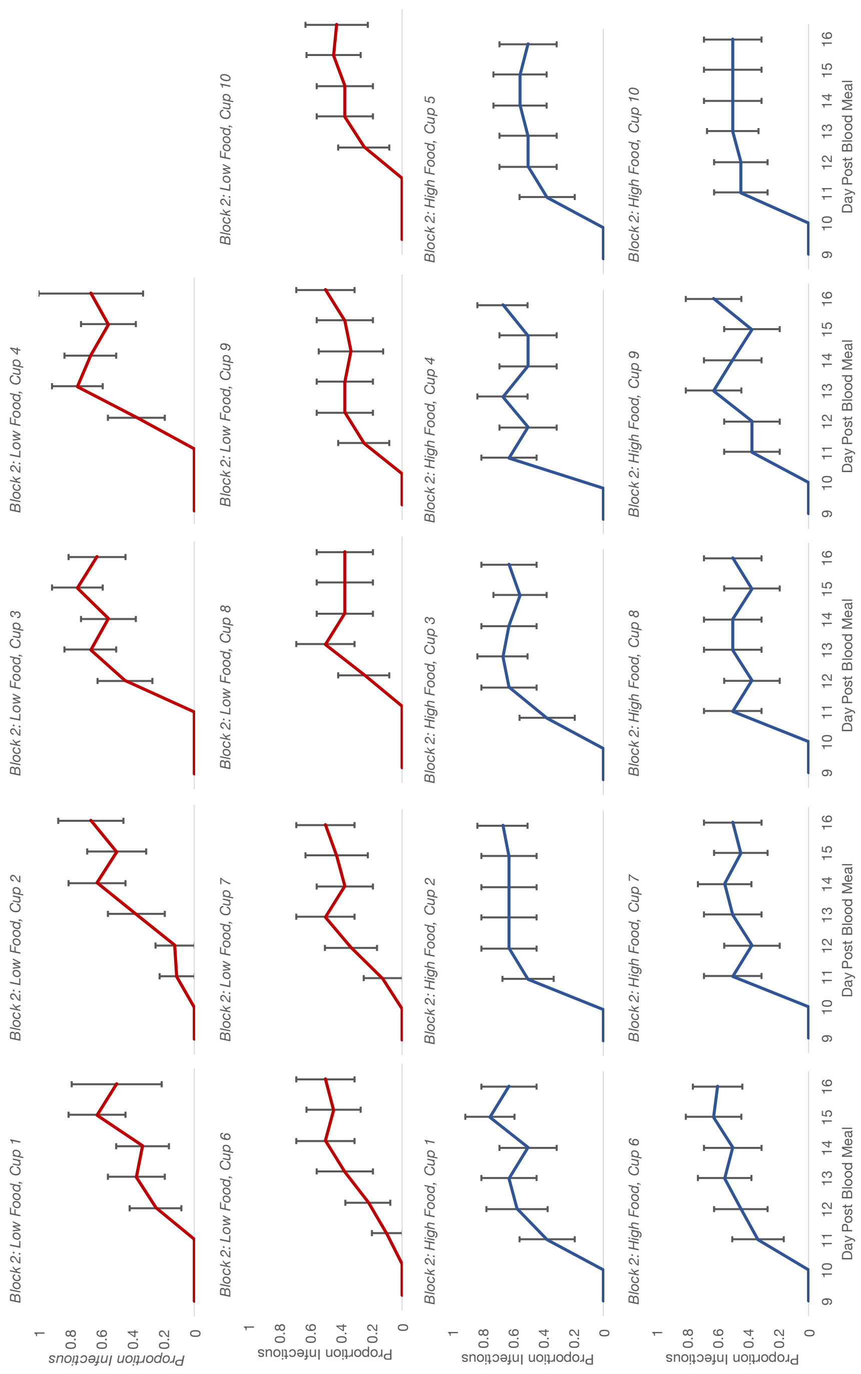

Figure S8. Dynamics of the change in proportion of infectious mosquitoes over time for each replicate cup in experimental block 2. In the low food group, replicate cup four was discarded, as the sugar cotton ball was mistakenly replaced with a water cotton ball post blood-meal, causing high mortality that would not allow for enough dissections over the time period of days 9-16 to be comparable to other cups within the experiment.

SUPPLEMENTARY TABLES

Supplementary Table S1.

	Oocyst Prevalence (n=210)			
**factor**	**estimate**	**std. error**	**z-value**	**pr(>|z|)**
intercept	**-0.483**	**0.218**	**-2.212**	**0.027**
treatment	**0.407**	**0.08**	**5.077**	**<0.001**
block	**-0.168**	**0.079**	**-2.117**	**0.034**
day	-0.012	0.023	-0.53	0.596

Table S1. Generalized linear mixed effects model output for oocyst prevalence. Bold indicates significance at α=0.05. Model fit assessed by deviance per degrees of freedom (=3.133).

Supplementary Table S2.

	Oocyst Intensity (n=210)			
**factor**	**estimate**	**std. error**	**df**	**p-value**
intercept	1.205	0.886	171	0.176
treatment	**8.495**	**1.247**	**31**	**<0.001**
block	0.04	0.551	31	0.943
day	-0.032	0.115	171	0.78
treatment x block	**-3.738**	**0.769**	**31**	**<0.001**
treatment x day	**-0.772**	**0.161**	**171**	**<0.001**
block x day	0.044	0.071	171	0.542
treatment x block x day	**0.369**	**0.099**	**171**	**<0.001**

Table S2. Linear mixed effects model output for oocyst intensity. Bold indicates significance at α=0.05. Model fit assessed by deviance per degrees of freedom (=1.944).

Supplementary Table S3.

**Model**	**Factors**	**df**	**AIC**	**ΔAIC**
gompertz	*food + block*	4	12438.49	0
gompertz2	*food*	3	12448.36	9.87
gompertz3	*block*	3	12577.58	139.09
weibull	*food + block*	4	12603.27	164.78
weibull2	*food*	3	12614.84	176.35
weibull3	*block*	3	12730.38	291.89

Table S3. AIC scores for each constructed survival curve model.

Supplementary Table S4.

**Designation**	**Effect**	**Parameter**
x	*treatment*	*g (asymptote)*
y	*treatment*	*k (rate)*
z	*treatment*	*tm (inflection)*
Q	*block*	*g*
R	*block*	*k*
S	*block*	*tm*
h	*treatment x block*	*g*
i	*treatment x block*	*k*
j	*treatment x block*	*tm*

Table S4. Term designations for each treatment, block or treatment x block effect oneach parameter of the intial EIP binary logistic model. Each candidate model contained the original terms g, k, and t_m.

Supplementary Table S5.

Model	df	AIC	ΔAIC
m.xyzQRSh	11	-595.52	0
m.xyzQRhi	11	-594.79	0.73
m.xyzQRShj	12	-593.74	1.78
m.xyzQRh	10	-593.59	1.93
m.xyzQSh	10	-592.45	3.07
m.xyzQRShij	13	-592.03	3.49
m.xyzQShj	11	-590.47	5.05
m.xyzQh	9	-589.09	6.43
m.xzQSh	9	-577.51	18.01
m.xzQShj	10	-575.62	19.90
m.xzQh	8	-574.97	20.55
m.xyzQRS	10	-551.84	43.68
m.xyzQR	9	-550.67	44.85
m.xyz	7	-534.95	60.57
m.xyzQ	8	-533.95	61.57
m.xzS	7	-521.75	73.77
m.xzQS	8	-521.41	74.11
m.xz	6	-521.22	74.30
m.xzQ	7	-519.69	75.83
m.z	5	-446.14	149.38
m.x	5	-445.39	150.13
m.zQ	6	-444.76	150.76
m.xQ	6	-443.55	151.97
m0 (null)	4	-317.19	278.33

Table S5. Competing models listed in order of least to greatest AIC values. Models are named according to terms specified in table S4.
